# Quantitative mapping of the per‐axon diffusion coefficients in brain white matter

**DOI:** 10.1002/mrm.25734

**Published:** 2015-05-13

**Authors:** Enrico Kaden, Frithjof Kruggel, Daniel C. Alexander

**Affiliations:** ^1^ Centre for Medical Image ComputingUniversity College LondonUK; ^2^ Department of Biomedical EngineeringUniversity of CaliforniaIrvineCaliforniaUSA

**Keywords:** per‐axon diffusion coefficients, fiber orientation distribution, powder average, spherical mean technique, microscopic diffusion anisotropy, diffusion MR imaging

## Abstract

**Purpose:**

This article presents a simple method for estimating the effective diffusion coefficients parallel and perpendicular to the axons unconfounded by the intravoxel fiber orientation distribution. We also call these parameters the per‐axon or microscopic diffusion coefficients.

**Theory and Methods:**

Diffusion MR imaging is used to probe the underlying tissue material. The key observation is that for a fixed *b*‐value the spherical mean of the diffusion signal over the gradient directions does not depend on the axon orientation distribution. By exploiting this invariance property, we propose a simple, fast, and robust estimator of the per‐axon diffusion coefficients, which we refer to as the spherical mean technique.

**Results:**

We demonstrate quantitative maps of the axon‐scale diffusion process, which has factored out the effects due to fiber dispersion and crossing, in human brain white matter. These microscopic diffusion coefficients are estimated in vivo using a widely available off‐the‐shelf pulse sequence featuring multiple *b*‐shells and high‐angular gradient resolution.

**Conclusion:**

The estimation of the per‐axon diffusion coefficients is essential for the accurate recovery of the fiber orientation distribution. In addition, the spherical mean technique enables us to discriminate microscopic tissue features from fiber dispersion, which potentially improves the sensitivity and/or specificity to various neurological conditions. Magn Reson Med, 2015. **Magn Reson Med 75:1752–1763, 2016. © 2015 The Authors. Magnetic Resonance in Medicine published by Wiley Periodicals, Inc.**

## INTRODUCTION

Today diffusion MR experiments are the method of choice for assessing the microscopic fiber anatomy noninvasively. The measured signal is sensitive to tissue properties in the range of few micrometers, such as the axon caliber, the degree of myelination, and the interaxonal space, averaged over a large population of microenvironments with potentially complex orientation distribution. The past years have seen considerable effort go into the development of biophysical models for estimating these structural tissue features, for example, the AxCaliber framework 1, 2 and the ActiveAx technique 3. A limitation of the previous studies is that the intravoxel axon population is assumed to consist of a single fiber bundle and that the axons within this bundle have the same orientation, which is typically not the case. Even in the corpus callosum the directional architecture is far from homogeneous 4, 5. The transcallosal fibers do not only link homotopic brain regions, but also heterotopic cortical areas, thus do not run parallel to each other, ultimately leading to complex orientation distributions 6. Furthermore, the presence of axon undulation, which is supposed to cope with mechanical tension such as pulsation, in other brain regions with eye movement and locomotion 7, gives rise to significant orientation dispersion, whose potential effect on the diffusion signal was recently demonstrated in a simulation study 8. Lastly, the callosal fibers may be arched on the millimeter voxel scale, in many instances apparent in the midsagittal plane.

To address the directional heterogeneity of white matter, we first model the diffusion signal of a small fiber segment up to its orientation. The spherical convolution of this impulse response function with the axon orientation distribution then yields the MR signal observable on the voxel scale 9. Based on a parametric spherical deconvolution approach 10, the assumption of a single fiber orientation that underpins previous methods was recently relaxed by allowing a Watson density of axon orientations to describe fiber dispersion 11, 12. However, the NODDI technique models only single fiber bundles even though strong evidence suggests that the majority of white matter regions features multiple fiber bundles crossing within the voxels 10, 13, 14, 15. Another potential limitation of NODDI and related methods is the assumption that the diffusion coefficient parallel to the fibers is fixed and known. Similarly, Jespersen et al. 16 developed a nervous tissue model to estimate the per‐axon/dendrite diffusion coefficients, which is also based on a mathematical model of the fiber orientation distribution, namely a spherical harmonic representation. This postmortem study of neonatal baboon brain, which used an MR dataset with a high signal‐to‐noise ratio (SNR), truncated the harmonic series expansion at an unduly low level to minimize the number of model parameters.

Alternatively, we may use a complex gradient sequence to recover the per‐axon diffusion coefficients in the presence of fiber dispersion and crossing. Double or multiple pulsed‐gradient‐field experiments 17, 18, 19, which encode Brownian motion with different gradient directions in two or more time periods, can detect microscopic diffusion anisotropy. See also Refs. ( 20 and 21 for two comprehensive reviews. Various anisotropy metrics, such as compartment eccentricity 22, were proposed, which are estimated from the signal differences we observe in dependence on the angle(s) between two (or more) gradient directions. A recent in vivo study 23 mapped microscopic anisotropy indices in human brain white matter, which are invariant with respect to the predominant fiber orientation. Furthermore, isotropic diffusion weighting based on magic‐angle *q*‐vector spinning 24 was proposed to factor out the effects due to orientation dispersion and to estimate the microscopic fractional anisotropy. To date, however, these nonconventional pulse sequences are not provided by the scanner vendors. The echo time is typically much longer than 100 ms, thus deteriorating the SNR and/or spatial resolution. A dense sampling of the high‐dimensional measurement space is required, especially for arbitrary, not necessarily uniform orientation distributions, which results in a long acquisition time that makes clinical usage rather difficult.

In this article, to factor out the effects due to fiber dispersion and crossing ubiquitous in brain white matter, we shall use neither prior knowledge about the fiber orientation distribution (e.g., Dirac measure, single Watson density, spherical‐harmonics model) nor complex diffusion sequences with multiple gradient pulses and magic‐angle spinning waveforms. Rather, the method we propose to quantify the per‐axon diffusion process and henceforth call the spherical mean technique (SMT) is based on the insight that for a fixed *b*‐value the spherical mean of the diffusion signal over the gradient directions does not depend on the fiber orientation distribution. In particular, the mean signal is only a function of the diffusion signal of individual axons. This approach is closely related to the powder average, which can be performed in two different ways, that is, by mixing the sample or by measuring the signal from all directions while keeping the other sequence parameters, hence the diffusion weighting factor, constant 25, 26, 27, 28. The presented technique can be easily adopted in the clinical domain, as it uses conventional pulse sequences featuring multiple *b*‐values and gradient directions. We have shown preliminary results in Ref. ( 29. The gain will be twofold. First, the specification of the impulse response function is essential for the quantitative recovery of the axon orientation distribution, which is used by tractography algorithms to reconstruct the fiber pathways in the brain 30, 31. Second, the per‐axon diffusion coefficients are simple‐to‐estimate markers sensitive to the underlying fiber microanatomy, such as the axon caliber, the degree of myelination, and the space between the fibers, that are not confounded by the orientational structure like the fractional anisotropy from the standard tensor model 32.

This article is organized as follows. We start with a brief introduction of the spherical convolution model 9, 13, 14. For general response functions it is shown that, when the *b*‐value is fixed, the mean signal over the gradient directions equals the spherical mean of the impulse response irrespective of the fiber orientation distribution. By exploiting this invariance property, we propose a simple, fast, and robust estimator of the microscopic diffusion process. The diffusion signal of individual axons is here modeled using a second‐order approximation. The Results section carries out a comprehensive simulation study and demonstrates the in vivo quantification of the per‐axon diffusion coefficients in the cerebral white matter without prior knowledge of the fiber orientation distribution. This report concludes with a discussion of SMT, including an outlook for future work.

## THEORY

### Biophysical Model

White matter tissue can be divided into an intracellular domain and extracellular space. The former component consists of axons, which may be strengthened by myelin sheath and are organized in bundles called fascicles, and glial cells, for example, oligodendrocytes, neurolemmocytes, and astrocytes, while the latter describes the space that separates the brain cells and is filled with interstitial fluid containing macromolecules of the extracellular matrix. Here, we start with a model for the diffusion signal of individual fibers. Consider the infinitesimal neighborhood of an axon oriented by the tangent vector $\omega \in S^{2}$, where $S^{2}=\{\omega \in {\mathbb{R}}^{3}:||\omega ||=1\}$ denotes the two‐dimensional unit sphere. If the axon pathways are sufficiently smooth, a fiber section in the micrometer range resembles an axially symmetric cylinder.

The diffusion signal of an axonal segment including its typical surrounding volume, which is always present and consists of glial cells and extracellular space, may be modeled by the impulse response function $h_{b}(g,\omega )$. b≥0 denotes the diffusion weighting factor and $g\in S^{2}$ the normalized gradient direction. Before proceeding, we present general properties of the impulse response. The diffusion signal *h_b_* of a small fiber section does not depend on its location within the voxel, as the MR experiment makes no attempt to encode this spatial information, and thus is voxel‐averaged. The per‐axon diffusion signal is a zonal function with $h_{b}(g,\omega )=h_{b}(Rg,R\omega )$ for all orientation‐preserving rotations R∈SO(3), where SO(3) denotes the special orthogonal group, which implies that *h_b_* depends only on the spherical distance 〈g,ω〉∈[−1,1] between any two points $g,\omega \in S^{2}$. We shall use both notations $h_{b}(g,\omega )=h_{b}(〈g,\omega 〉)$ interchangeably. The impulse response is antipodally symmetric, that is, $h_{b}(g,\omega )=h_{b}(-g,\omega )$ for all $g,\omega \in S^{2}$, and takes its values in the interval [0,1]. Henceforth, let us assume that the signal *h_b_* of an axonal segment is known up to its orientation $\omega \in S^{2}$.

The fiber orientation distribution $p:S^{2}\to [0,\infty ]$ quantifies the relative frequency of specific axon orientations within a fiber population. This density function is characterized by antipodal symmetry, that is, $p(\omega )=p(-\omega ),\;\omega \in S^{2}$, non‐negativity, that is, $p(\omega )\geq 0,\;\omega \in S^{2}$, and normalization, that is, $\int_{S^{2}}p(\omega )\;d\omega =1$ 15. The spherical convolution of *p* with the per‐axon diffusion signal *h_b_*,

(1) $$\frac{E_{b}(g)}{E_{0}}=\int_{S^{2}}h_{b}(g,\omega )\;p(\omega )\;d\omega ,$$

yields the observable MR signal on the voxel scale 9, 13, 14. $E_{b}(g)$ is the signal with the diffusion encoding b≥0 and $g\in S^{2}$, while *E*_0_ denotes the signal in the absence of diffusion weighting, which is required for normalizing the *T*_2_‐contrast. Finally, the diffusion signal $e_{b}(g)=E_{b}(g)/E_{0}$ is antipodally symmetric and takes its values in [0,1]. For a modern measure‐theoretic perspective we refer the reader to Kaden and Kruggel 33.

### Spherical Mean Technique

Thus far, we have assumed that the diffusion signal of a fiber segment is known, which is, however, not the case. To estimate the impulse response without prior knowledge about the tangential distribution of the axons, we consider the spherical mean of the diffusion signal over the gradient directions

(2) $${\bar{e}}_{b}=\frac{1}{4\pi }\int_{S^{2}}e_{b}(g)\;dg,$$

where all other sequence parameters, in particular the diffusion weighting factor b≥0, are fixed. The key insight is that the mean signal ${\bar{e}}_{b}$ is invariant with respect to the fiber orientation distribution. To see this, we first show that the spherical mean of the response function, that is, the diffusion signal of an axonal segment,

(3) $${\bar{h}}_{b}=\frac{1}{4\pi }\int_{S^{2}}h_{b}(g,\omega )\;dg$$

does not depend on the fiber orientation $\omega \in S^{2}$. For two arbitrarily chosen orientations $\omega_{1},\omega_{2}\in S^{2}$ we can always find an orientation‐preserving rotation R∈SO(3) with $\omega_{1}=R\omega_{2}$. Therefore, it holds

(4) $$\begin{array}{l} \frac{1}{4\pi }\int_{S^{2}}h_{b}(g,\omega_{2})\;dg\overset{\left(i\right)}{=}\frac{1}{4\pi }\int_{S^{2}}h_{b}(Rg,R\omega_{2})\;dg\\ =\frac{1}{4\pi }\int_{S^{2}}h_{b}(Rg,\omega_{1})\;dg\overset{\left(\mathrm{ii}\right)}{=}\frac{1}{4\pi }\int_{S^{2}}h_{b}(g,\omega_{1})\;dg, \end{array}$$

as (i) *h_b_* depends only on the inner product of its arguments and (ii) integration is translation‐invariant with respect to SO(3).

Next, we show the invariance property in generality. We substitute the spherical convolution model (1) into Eq. [2] and change the order of integration, which is justified according to Fubini's theorem, recalling that the response function $|h_{b}(g,\omega )|\leq 1$ is bounded for all $g,\omega \in S^{2}$ and the surface area of *S* ^2^ as well as the spherical integral $\int_{S^{2}}p(\omega )\;d\omega =1$ are finite. The inner integral in

(5) $${\bar{e}}_{b}=\int_{S^{2}}\left(\frac{1}{4\pi }\int_{S^{2}}h_{b}(g,\omega )\;dg\right)p(\omega )\;d\omega ={\bar{h}}_{b}\int_{S^{2}}p(\omega )\;d\omega$$

is not a function of *ω*, as demonstrated above, thus gives the spherical mean ${\bar{h}}_{b}$ of the response function, and our claim follows. In particular, the mean signal ${\bar{e}}_{b}$ for an arbitrary fiber orientation distribution equals the spherical mean ${\bar{h}}_{b}$ of the diffusion signal of a small axonal segment. Note that we have not used a particular impulse response, but only properties common to all response functions. This spherical mean lemma can be shown in a similar way for the measure‐theoretic approach 33. To compute the spherical mean, it is sufficient to calculate the mean signal of the impulse response whose orientation can be chosen arbitrarily, here for convenience $\omega ={\left(0,0,1\right)}^{t}\in S^{2}$. After a transformation into spherical coordinates and recalling the antipodal symmetry of *h_b_*, the mean diffusion signal takes the form

(6) $${\bar{e}}_{b}=\int_{0}^{\pi /2}h_{b}(\cos(\theta ))\sin(\theta )\;d\theta ,$$

which is easy to compute. The insight that for a specified *b*‐value ${\bar{e}}_{b}$ is fully determined by the response function will enable us to infer the axon microanatomy without any information about the directional tissue architecture. We call this method the spherical mean technique (SMT).

### Second‐Order Approximation

In this work, we model the diffusion signal of an axonal segment using a second‐order approximation 26, which may be written as

(7) $$h_{b}(g,\omega )=\underset{\text{longitudinal}}{\underset{︸}{\exp\left(-b{〈g,\omega 〉}^{2}\lambda_{∥}\right)}}\;\underset{\text{transverse}}{\underset{︸}{\exp\left(-b(1-{〈g,\omega 〉}^{2})\lambda_{⊥}\right)}}.$$

This axially symmetric microscopic tensor model is parametrized by $\lambda_{∥}$ and $\lambda_{⊥}$, which denote the effective diffusion coefficients parallel to a small fiber section and perpendicular to it, respectively. $\lambda_{∥}$ and $\lambda_{⊥}$ quantify the voxel‐averaged diffusion process inside the axon and in its characteristic vicinity with the constraint $0\leq \lambda_{⊥}\leq \lambda_{∥}\leq \lambda_{\text{free}}$ because the axonal membranes perpendicular to the fiber axis $\omega \in S^{2}$ form the major barriers that confine Brownian motion of water molecules. The upper bound $\lambda_{\text{free}}$ is given by the bulk diffusivity, which is circa $3.05\;{\mu m}^{2}/\text{ms}$ at 37°C 34. Equation (7) fulfils the general properties of an impulse response presented above. Eventually, the spherical mean of the diffusion signal with the parametric response model (7) reads

(8) $$\begin{array}{l} {\bar{e}}_{b}(\lambda_{∥},\lambda_{⊥})=\exp{\left(-b\lambda_{⊥}\right)}_{1}F_{1}(1/2;3/2;-b(\lambda_{∥}-\lambda_{⊥}))\\ =\exp(-b\lambda_{⊥})\frac{\sqrt{\pi }\text{erf}(\sqrt{b(\lambda_{∥}-\lambda_{⊥})})}{2\sqrt{b(\lambda_{∥}-\lambda_{⊥})}}, \end{array}$$

where ${}_{1}F_{1}$ denotes the confluent hypergeometric function and erf is the error function. Equation [8] has been derived before 10, 14, 35, but was used in a different context, that is, for the recovery of the fiber orientation distribution.

## METHODS

### Experiment Design

The diffusion data analyzed in the present study were kindly provided by the Human Connectome Project, WU‐Minn Consortium (HCP Lifespan pilot data, Phase 1a, Washington University, released August 2014, available online at http://www.humanconnectome.org). The dataset was acquired on a Siemens 3 T Skyra MRI scanner equipped with a 32‐channel phased‐array head coil and a customized SC72 gradient insert featuring a maximum gradient strength of 100 mT/m 36. A spin‐echo Stejskal–Tanner sequence measured two *b*‐shells of about 1000 and 2500 s/mm^2^ with 76 and 75 gradient directions, respectively, which together with their antipodal points are uniformly distributed on the sphere 37. SMT requires at least two non‐zero diffusion weighting factors for the quantification of the per‐axon diffusion coefficients $\lambda_{∥}$ and $\lambda_{⊥}$.

Note that $\lambda_{∥}$ and $\lambda_{⊥}$ do not depend only on the microscopic diffusion process, for example, bulk diffusivity, fiber microgeometry, and observation time, but also on the MR experiment, that is, the temporal profile of the diffusion encoding gradients. In particular, two different pulse sequences with identical *b*‐value may give rise to different effective diffusion coefficients. Therefore, it is imperative that the sequence timing, that is, the temporal profile of the diffusion sensitizing gradients, is kept fixed, here with echo time of 74.8 ms and repetition time of 3.67 s, so that the same diffusion propagator is observed throughout the scan. Only the magnitude |G|, hence the diffusion weighting factor *b*, and the direction $g=G/|G|\in S^{2}$ of the time‐dependent diffusion encoding gradients *G*(*t*) are altered. Additionally, 10 images without diffusion weighting were measured, which are evenly distributed across the experiment.

The spin‐echo echo‐planar scan with nominal flip angle of $78^{{}^{\circ}}$ and refocusing flip angle of $160^{{}^{\circ}}$ was performed using a multiband sequence 38, 39, 40 with slice acceleration factor of 3. The diffusion data were acquired with in‐plane phase encoding in both right‐to‐left and left‐to‐right directions. The measurement of 93 slices with 1.5 mm thickness and a 140 × 120 image matrix with a field of view of 210 × 180 mm^2^ (in readout and phase‐encoding direction, respectively) covered the whole brain, resulting in an isotropic voxel resolution of 1.5 mm. The acquisition time was circa 36 min. After unaliasing the simultaneously acquired slices channel by channel 38, 39, 40, SENSE1 multiple‐coil combination was applied 41 and the magnitude signal was stored. The dataset used in the study came from an adult male in the age group between 25 and 35 years.

### Data Preprocessing

The image data were preprocessed using HCP's Minimal Preprocessing Pipeline 42, 43. This pipeline starts with intensity normalization across the diffusion scan based on the zero *b*‐value images. The susceptibility‐induced distortions are eliminated using the two images acquired with reversed phase‐encoding polarities 44, 45. Further, the dataset is corrected for eddy‐current artefacts and subject motion, as implemented in the FMRIB Software Library 46. All corrections are performed in a single resampling step. In this work, we analyze the diffusion‐weighted images in the native measurement space. The brain is extracted from the dataset 47 and the image background is masked.

As the MR signal was combined with SENSE1 from the multiple receive coils, the noise regime of the magnitude signal, although data preprocessing may alter its characteristics to a certain extent, is well described by a Rician distribution R(E,ς) 48. The probability density function is defined as

(9) $$f_{R}(S;E,\varsigma )=\frac{S}{\varsigma^{2}}\exp\left(-\frac{S^{2}+E^{2}}{2\varsigma^{2}}\right)I_{0}\left(\frac{SE}{\varsigma^{2}}\right),$$

where S∈[0,∞) is the measured noisy signal, E≥0 denotes the true magnitude signal, ς>0 characterizes the noise level, and *I*_0_ is the zeroth‐order modified Bessel function of the first kind. The mean of the Rician distribution is higher than the true signal especially for low SNRs. To minimize potential effects of this noise‐induced bias, we adjust the measured signal as follows

(10) $$\hat{E}=\arg\underset{E\geq 0}{\min}{\left(S-\sqrt{\frac{\pi \varsigma^{2}}{2}}L_{1/2}\left(-\frac{E^{2}}{2\varsigma^{2}}\right)\right)}^{2},$$

where $\hat{E}$ denotes the adjusted signal, the second term on the right‐hand side is the mean of the Rician distribution, and *L_n_* stands for the *n*th Laguerre polynomial. ς is computed as the median of the noise level estimated voxel by voxel from the 10 images without diffusion weighting using a maximum‐likelihood approach 49, noting that the spatial noise distribution is approximately uniform over the cerebral white matter (with an SNR of about 17.5) in the dataset analyzed here.

### Sample Mean Estimator

Next, we will quantify the per‐axon diffusion coefficients without prior knowledge of the fiber orientation distribution. To estimate the spherical mean signal (2), we average the Rician‐noise adjusted diffusion signals acquired with pairwise different gradient directions for each diffusion weighting factor *b* separately. These signals have been normalized by the *T*_2_‐contrast before, using the MR signal in the absence of diffusion encoding. The imaging gradients and nonlinearities in the gradient field give rise to small spatial variations in the *b*‐value. In the following, we perform the calculations with the average weighting factor per *b*‐shell and use the nominal values, in this study 1000 and 2500 s/mm^2^, to refer to them. The sample mean ${\hat{e}}_{b}$ yields a close approximation of the spherical mean ${\bar{e}}_{b}$ of the diffusion signal when the gradient directions and their antipodal points are uniformly distributed on the sphere. For nonuniform directional gradient schemes the mean signal may be estimated using a reproducing kernel Hilbert space technique 50.

In a second step, the model parameters of the impulse response are determined, here the microscopic diffusion coefficients $\lambda_{∥}$ and $\lambda_{⊥}$ parallel and perpendicular to an axonal segment. This parametric estimation of the response function (7) is performed by using a constrained least‐squares approach

(11) $${\hat{e}}_{b}$$

that fits the mean signal estimates ${\hat{e}}_{b_{i}}$ to the expected spherical mean ${\bar{e}}_{b_{i}}(\lambda_{∥},\lambda_{⊥})$ formulated in Eq. [8] for a given set of *n* diffusion weighting factors *b_i_* with i={1,…,n}. The per‐axon diffusion coefficients $\lambda_{∥}$ and $\lambda_{⊥}$ are estimated subject to $0\leq \lambda_{⊥}\leq \lambda_{∥}\leq \lambda_{\text{free}}$, where $\lambda_{\text{free}}$ denotes the free water diffusivity. Therefore, the recovered diffusion parameters are ensured to lie within a physically meaningful range. SMT requires two or more *b*‐shells (i.e., n≥2) as otherwise the estimation problem (11) is underdetermined.

## RESULTS

### Simulations

**Figure 1:**
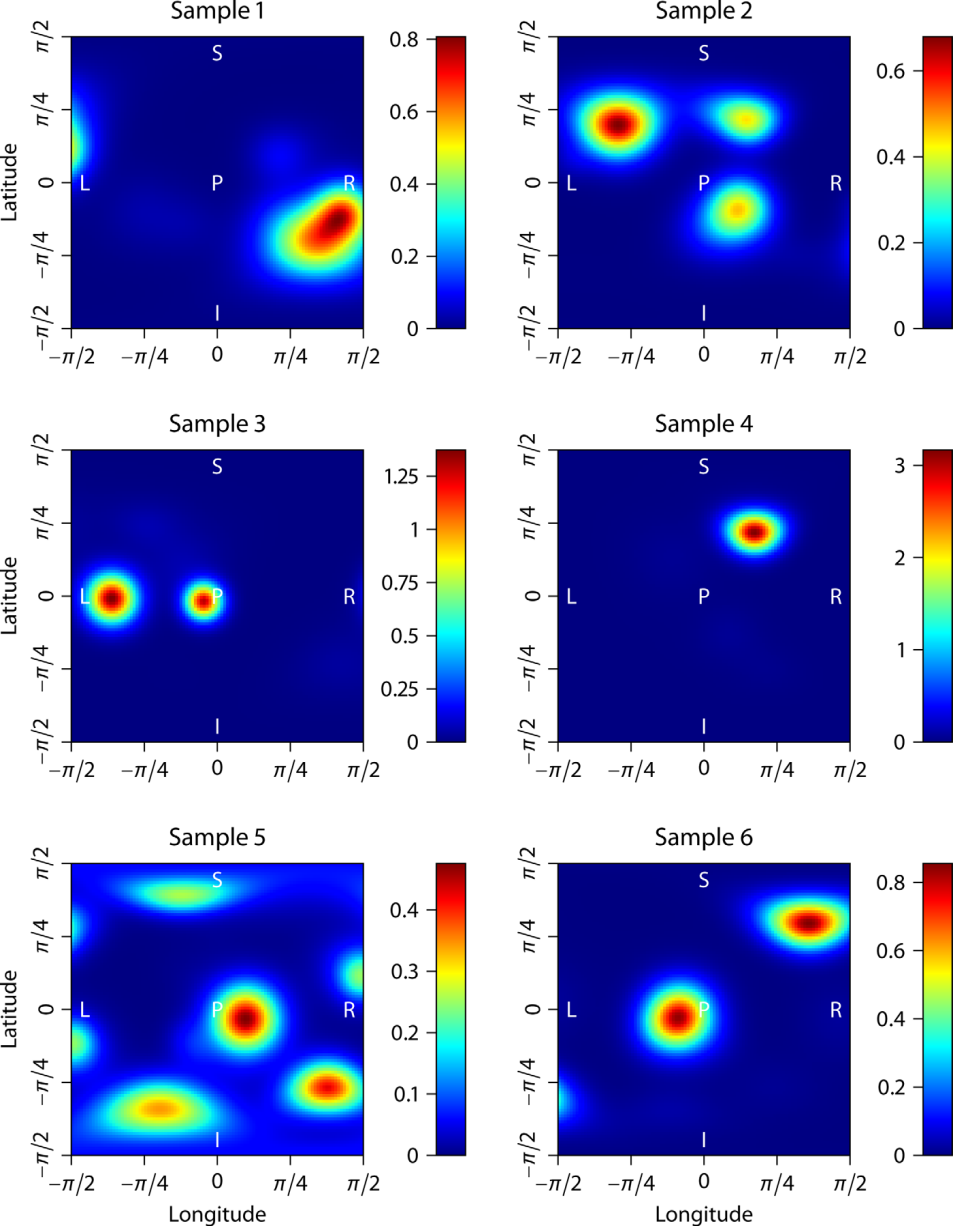
Simulation of the fiber orientation distribution using a Dirichlet process mixture with bipolar Watson kernel, which is shown for six samples. The spherical functions are depicted in polar coordinates and, because these functions are antipodally symmetric, it is sufficient to display them in one hemisphere. Abbreviations: left (L), right (R), inferior (I), superior (S), anterior (A), posterior (P).

To demonstrate the reliability of SMT, we simulate fiber orientation distributions that closely resemble the tissue geometry of white matter. Here, we use a Dirichlet process mixture with bipolar Watson kernel 33 to draw random spherical density functions. In the stick‐breaking representation, this infinite mixture model takes the form

(12) $$p(\omega )=\sum_{i=1}^{\infty }\pi_{i}\frac{\exp(\kappa_{i}{〈\omega ,\nu_{i}〉}^{2})}{4\pi_{1}F_{1}(1/2;3/2;\kappa_{i})},$$

where $\pi_{i}=X_{i}\prod_{j=1}^{i-1}\left(1-X_{j}\right)$ are random weights. $X_{i}∼Be(1,\alpha )$ are governed by a Beta distribution with α=1.5; $\nu_{i}∼U_{S^{2}}$ and $\kappa_{i}∼IG(\alpha_{\kappa },\beta_{\kappa })$ are drawn from the spherical uniform distribution and inverse Gamma density with hyperparameters $\alpha_{\kappa }=4.71$ and $\beta_{\kappa }=57.1$ (with maximum density at $\kappa_{\text{mode}}=10$ and $P[\kappa \leq 50\;|\;\alpha_{\kappa },\beta_{\kappa }]=0.99$), respectively. Under the topology of weak convergence the Dirichlet process mixture includes all fiber orientation distributions in its closure 51. It is easy to see that the characteristic properties of *p*, that is, antipodal symmetry, non‐negativity, and normalization, are fulfilled. Figure 1 depicts six samples of fiber orientation densities drawn from this stochastic process, illustrating that the synthetic distributions include a broad range of axon dispersion and crossing. The spherical convolution of *p* with the impulse response (7) yields the diffusion signal, which is given by Kaden and Kruggel 33. For our simulation experiments the per‐axon diffusion coefficients are set to $\lambda_{∥}=2.5$ and $\lambda_{⊥}=0.1\;\mu m^{2}/\text{ms}$ (cf. Fig. 8).

**Figure 2:**
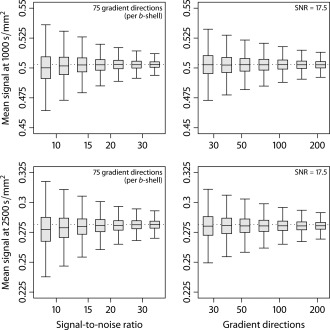
Estimation accuracy of the mean diffusion signal ${\bar{e}}_{b}$ for the *b*‐values of 1000 and 2500 s/mm^2^ using the sample mean estimator. The left panel depicts box‐and‐whisker plots (with 1.5 times the interquartile range) for the acquisition protocol and various SNRs. In the right column, the estimated spherical mean of the diffusion signal is shown for different numbers of gradient directions. The true mean signals (dotted lines) read {0.503,0.282} for {1000,2500} s/mm^2^, respectively.

We examine the accuracy of the SMT estimator using simulated data, which were generated by the fiber dispersion model and then disturbed by Rician noise. 5000 trials each were run to investigate the estimation error of the mean diffusion signal and per‐axon diffusion coefficients under various scenarios after adjustment for the Rician noise bias. Figure 2 depicts box‐and‐whisker plots (with 1.5 times the interquartile range) of the estimated spherical mean for the *b*‐values of 1000 and 2500 s/mm^2^ with respect to SNR (left column), using the measurement protocol of the dataset analyzed in this work, and different numbers of gradient directions per diffusion weighting factor. The fixed parameter is indicated in a corner of the diagrams.

**Figure 3:**
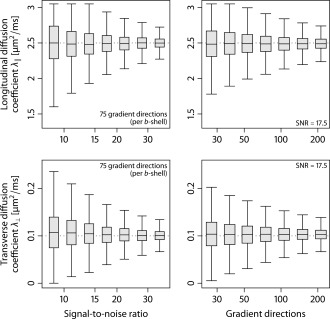
Estimation accuracy of the per‐axon diffusion coefficients $\lambda_{∥}$ and $\lambda_{⊥}$. In the left column, the estimated parameters are shown for the measurement protocol and various SNRs. The right column depicts box‐and‐whisker plots (with 1.5 times the interquartile range) for different numbers of gradient directions per *b*‐value. The true diffusion coefficients (dotted lines) were set to $\lambda_{∥}=2.5$ and $\lambda_{⊥}=0.1\;\mu m^{2}/\text{ms}$.

Figure 3 displays the estimated diffusion coefficients $\lambda_{∥}$ and $\lambda_{⊥}$ as a function of SNR (left column), using the acquisition protocol, and the number of gradient directions measured for each *b*‐value of 1000 and 2500 s/mm^2^. The box‐and‐whisker diagrams demonstrate that the variance of the estimator decreases as SNR and/or the number of gradient directions per *b*‐value (together with their antipodal points uniformly distributed on the sphere) increase. This average‐case study over density functions drawn from a Dirichlet process mixture suggests that SMT is a robust estimator of $\lambda_{∥}$ and $\lambda_{⊥}$. The computer simulations also show that adverse effects due to the Rician noise regime are removed to a large extent. To summarize, a moderate number of diffusion encoding gradients appears to be sufficient to recover the per‐axon diffusion process regardless of the fiber orientation distribution.

### Data Analysis

**Figure 4:**
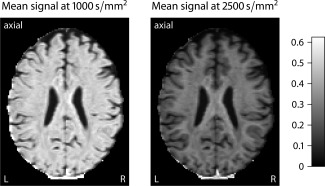
These plots depict, in the axial plane, the spherical mean ${\bar{e}}_{b}$ of the diffusion signal for the *b*‐values of 1000 and 2500 s/mm^2^ using the sample mean estimator.

**Figure 5:**
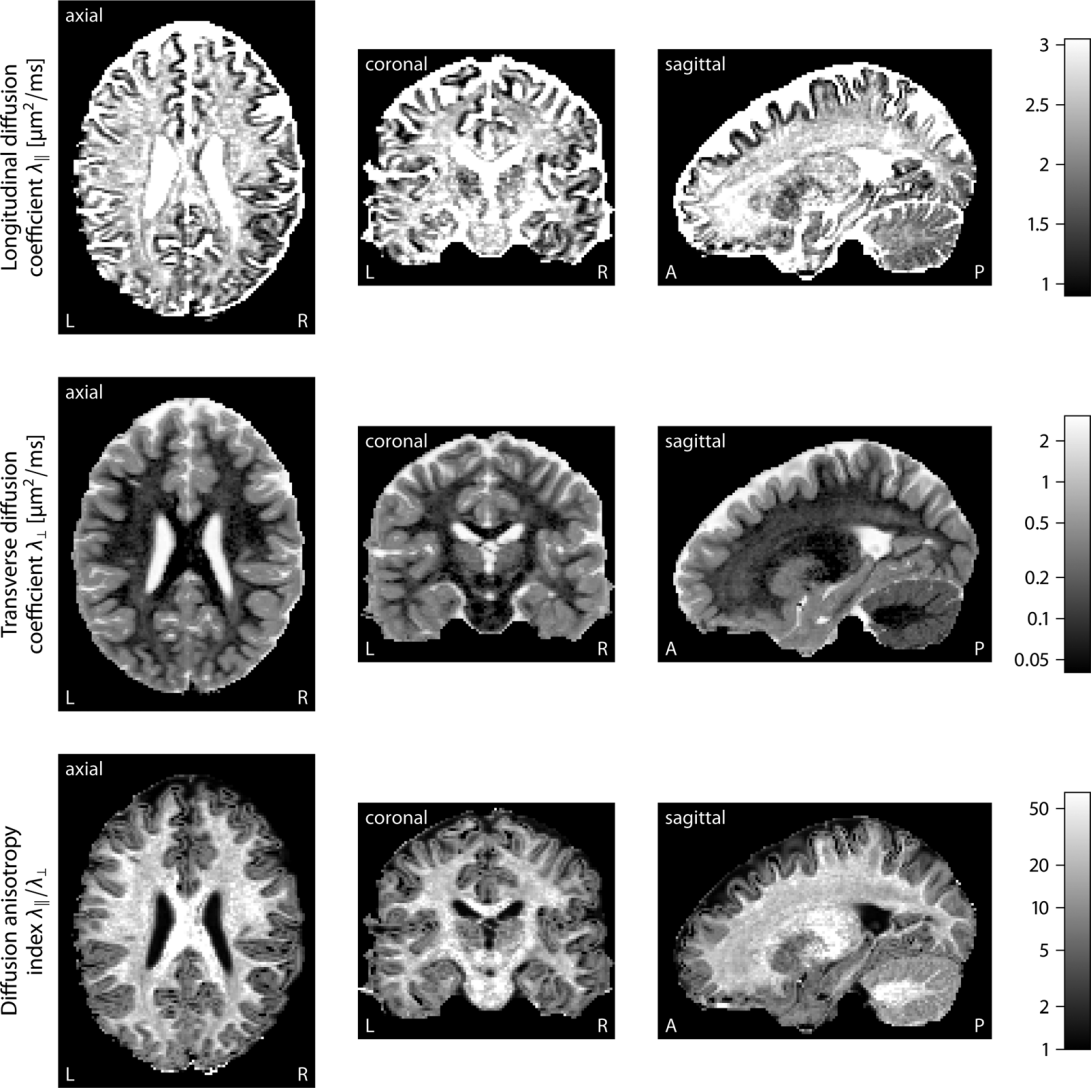
The upper two rows map the longitudinal and transverse microscopic diffusion coefficients $\lambda_{∥}$ and $\lambda_{⊥}$ of the axons, shown in the axial, coronal, and sagittal plane (from left to right). In the bottom panel, the anisotropy index $\lambda_{∥}/\lambda_{⊥}$ of the per‐axon diffusion process is plotted. The estimated parameters have factored out the effects due to the intravoxel fiber orientation distribution.

We continue with the in vivo quantification of the per‐axon diffusion process in a healthy volunteer. Prior to this, intermediate results are shown. Figure 4 maps the spherical mean ${\bar{e}}_{b}$ of the diffusion signal for each *b*‐value of 1000 and 2500 s/mm^2^. The voxel‐by‐voxel vector of the mean signals, which is a function of the axon microanatomy but without the effects due to fiber dispersion and crossing, is an important biomarker in its own right. Figure 5 plots, in the axial, coronal, and sagittal plane (from left to right), the diffusion coefficients $\lambda_{∥}$ and $\lambda_{⊥}$ parallel to individual axons and perpendicular to them (first and second row) in the brain white matter, noting that SMT has not made any assumptions about the intravoxel fiber orientation distribution. The bottom row of the figure shows the per‐axon anisotropy index, which is defined as the ratio $\lambda_{∥}/\lambda_{⊥}$ of the longitudinal and the transverse diffusion coefficient. The estimated quantities, which reflect the microscopic diffusion process inside the axons and in their typical neighborhood, are average values over the fiber population in the displayed voxel.

Figure 5 demonstrates that $\lambda_{∥}$ and $\lambda_{⊥}$ vary in the human brain, providing evidence that the axonal microenvironments are heterogeneous beyond their tangential distribution. For instance, the transverse diffusion coefficient $\lambda_{⊥}$ is lower in the corpus callosum compared to other white matter regions, which might be due to the converging pattern of the callosal fibers that results in a reduction of the interaxonal space accompanied with a higher axon density. Furthermore, our data analysis suggests that the longitudinal diffusion coefficient $\lambda_{∥}$ is significantly higher than previous studies have suggested. This numerical underestimation might adversely affect the estimation of structural features such as the axon caliber or fiber density. Supporting Information Figure S1 shows additional results for subjects in the same age group from the HCP Lifespan data. The estimated diffusion coefficients for the ventricular system filled with cerebrospinal fluid approaches the bulk diffusivity of free water. Lastly, $\lambda_{∥}$ and $\lambda_{⊥}$ are distinct from the axial and radial diffusion parameters provided by the classical tensor model 32, which are influenced by fiber dispersion and crossing.

The microscopic diffusion process may be condensed into the per‐axon mean diffusivity, which is defined as $\bar{\lambda }=(\lambda_{∥}+2\lambda_{⊥})/3$, and the per‐axon fractional anisotropy, which is given by

(13) $$\text{FA}=\sqrt{\frac{3}{2}\frac{{\left(\lambda_{∥}-\bar{\lambda }\right)}^{2}+2{\left(\lambda_{⊥}-\bar{\lambda }\right)}^{2}}{\lambda_{∥}^{2}+2\lambda_{⊥}^{2}}}.$$

**Figure 6:**
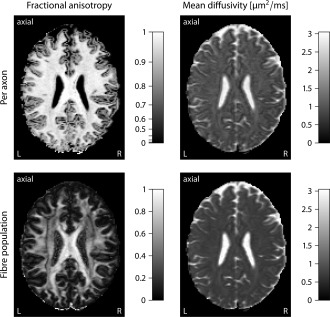
These plots map the fractional anisotropy (left) and mean diffusivity for individual axons (top) and the entire fiber population, comparing SMT with the standard tensor model. In the upper row, the per‐axon diffusion process is shown, which does not depend on the fiber orientation distribution, while the bottom section summarizes the fiber‐population water diffusion in the displayed voxels.

Figure 6 maps the fractional anisotropy and mean diffusivity for individual axons (top) and the entire fiber population, comparing the proposed technique with the classical tensor model 32. The per‐axon fractional anisotropy, which has factored out the intravoxel fiber orientation distribution, exhibits rather high values close to one in white matter tissue, showing a strong directional preference $\lambda_{∥}\gg \lambda_{⊥}$ of the local water diffusion. In contrast, the fractional anisotropy of the standard tensor model is affected by the axon orientation distribution. For example, the centrum semiovale has occasionally a fractional anisotropy lower than 0.2, suggesting almost isotropic diffusion at the voxel‐resolution level. In fact, this brain region has a complex orientational structure with crossings of the pyramidal tract, the callosal fibers, and the superior longitudinal fasciculus 10, 35. The mean diffusivity of an axonal segment and the quantity derived from the classical tensor model share a similar image contrast, but from a theoretical viewpoint the per‐axon and fiber‐population mean diffusivities are in general two different indices.

**Figure 7:**
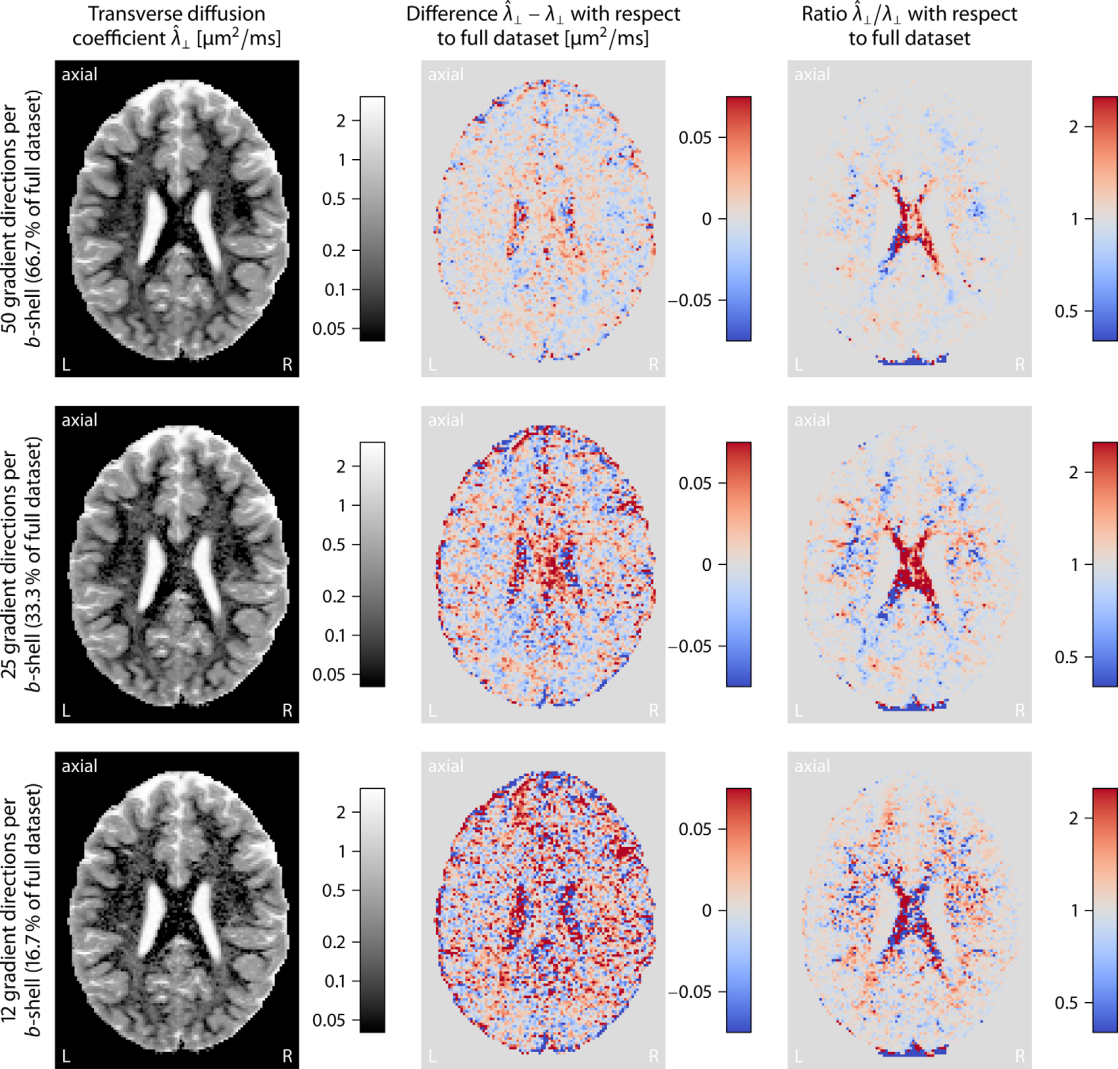
Estimation accuracy for 66.7%, 33.3%, and 16.7% subsets of gradient directions per *b*‐shell, here 1000 and 2500 s/mm^2^ (from top to bottom). The first column depicts the transverse per‐axon diffusion coefficient ${\hat{\lambda }}_{⊥}$. In the second and third columns, the difference ${\hat{\lambda }}_{⊥}-\lambda_{⊥}$ and the ratio ${\hat{\lambda }}_{⊥}/\lambda_{⊥}$ are shown with respect to the estimated diffusivity $\lambda_{⊥}$ in the full dataset (cf. Fig. 5).

**Figure 8:**
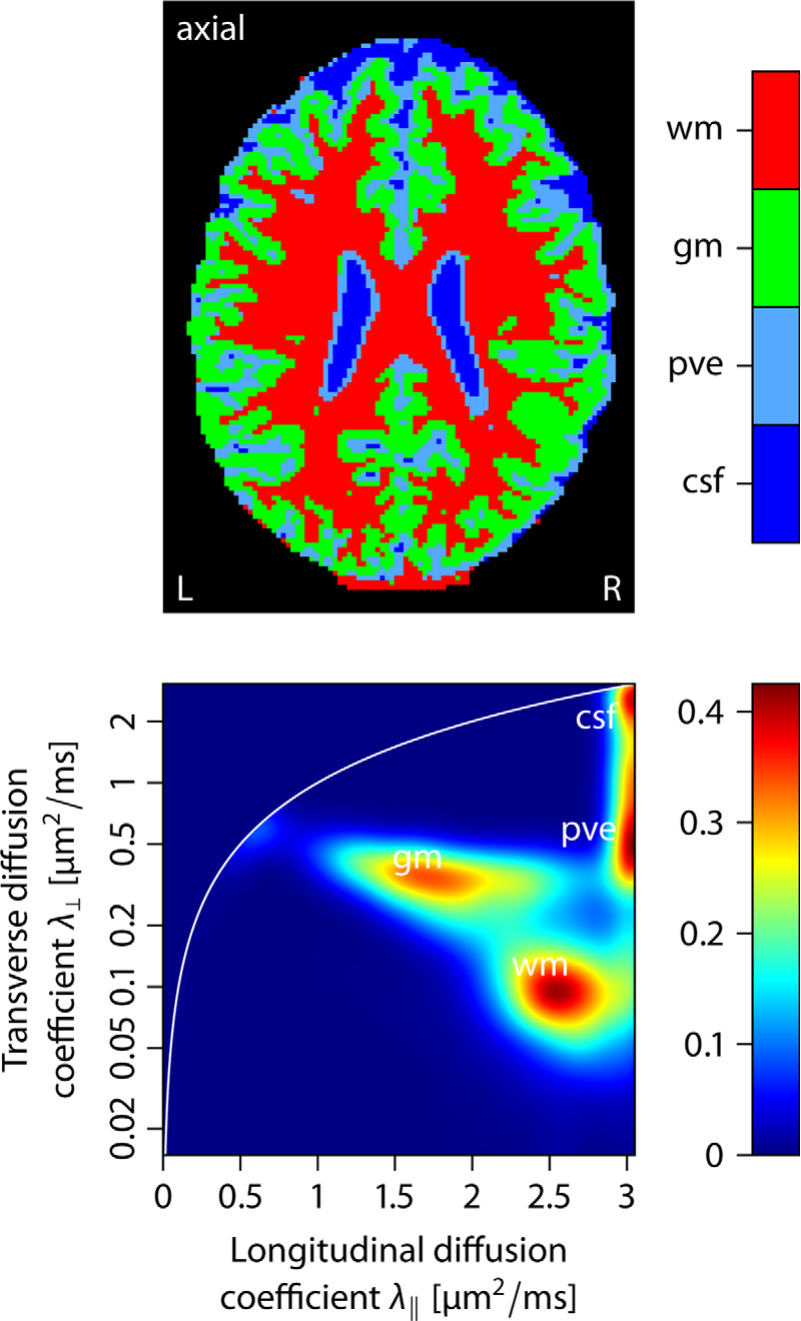
Density plot of the per‐axon diffusion coefficients exemplified for the axial slice shown in Figure 5. The white line marks isotropic microscopic diffusion with $\lambda_{∥}=\lambda_{⊥}$. In the lower diagram, we can identify four clusters. The upper image maps the compartment the displayed voxel is assigned to. Abbreviations: white matter (wm), grey matter (gm), partial volume effects (pve), cerebrospinal fluid (csf).

The diffusion‐encoding gradients were arranged in a way so that every subset of the first *n* directions, together with their antipodal points, are also uniformly distributed on the sphere 37. Figure 7 maps the transverse diffusion coefficient ${\hat{\lambda }}_{⊥}$ for subsets of 50, 25, and 12 gradient directions per *b*‐shell, here 1000 and 2500 s/mm^2^ (with scan times of circa 24, 12, and 6 min, respectively), as well as the difference ${\hat{\lambda }}_{⊥}-\lambda_{⊥}$ and the ratio ${\hat{\lambda }}_{⊥}/\lambda_{⊥}$ with respect to $\lambda_{⊥}$ estimated from the full dataset. This figure shows that the axon‐scale diffusion process can be recovered from data with a lower angular gradient resolution at the expense of a noisier appearance and a slight bias in the SMT estimates, demonstrating the clinical applicability of the developed imaging technique. Comparable results are obtained for the longitudinal diffusion coefficient $\lambda_{∥}$. Figure 8 depicts a density plot of the per‐axon diffusion coefficients, summarizing the results from the axial slice shown in Figure 5. This diagram suggests that there are four clusters of similar microscopic diffusivities, which can be associated with white matter, gray matter, cerebrospinal fluid, and partial volume effects. The latter voxels are composed of tissue that is contaminated with cerebrospinal fluid. The upper panel of this figure maps, in the axial plane, the compartment the displayed voxel is assigned to, providing a natural segmentation of the brain anatomy.

**Figure 9:**
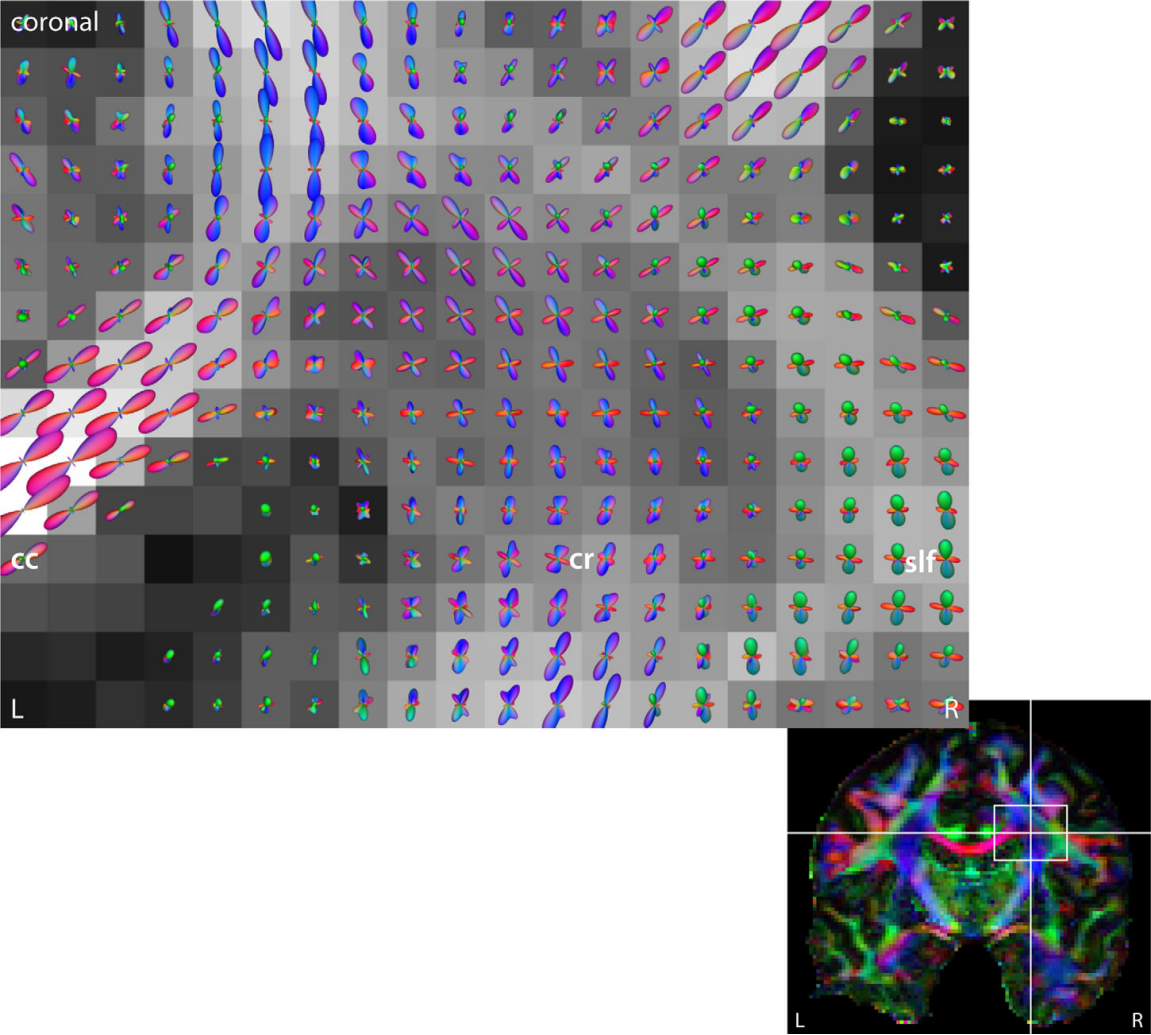
Fiber orientation field of the centrum semiovale in the right hemisphere, uncovering the radiation of the corpus callosum (cc), the corona radiata (cr), and their crossing (shown in the coronal plane). The spherical deconvolution of the axon orientation distribution is performed in a reproducing kernel Hilbert space 35, 50. The underlying map depicts the fractional anisotropy of the classical tensor model. Abbreviation: superior longitudinal fasciculus (slf).

Following the voxel‐by‐voxel estimation of the per‐axon diffusion coefficients $\lambda_{∥}$ and $\lambda_{⊥}$, we are able to recover the fiber orientation distribution from the diffusion measurements. Knowing the spatially varying response function enables us to solve the inverse problem accurately for the first time. To demonstrate this, we present results in Figure 9 using the spherical deconvolution in a reproducing kernel Hilbert space, which was proposed by Kaden et al. 35. This technique does not truncate the harmonic series expansion of the axon orientation distribution as in past studies 13, 14 and ensures all characteristic properties of the density function, namely its antipodal symmetry, non‐negativity, and normalization with one. The figure shows the reconstruction of the fiber orientation field in the centrum semiovale of the right hemisphere, which is estimated from all *b*‐shell data. The fiber orientation density *p* is visualized by the quasi‐spherical surface $S^{2}∋\omega \mapsto p(\omega )\omega \in {\mathbb{R}}^{3}$ with the following color encoding: Red indicates a left‐right orientation, green an anterior‐posterior direction, and blue a superior‐inferior orientation. Figure 9 exposes, in the coronal plane, the intermingling of the pyramidal tract with the radiating callosal fibers. Alternative deconvolution techniques which may be used were proposed in Refs. ( 10 and 33.

## DISCUSSION

It is difficult, if not impossible, to examine the axon microgeometry using markers that are affected by fiber dispersion and crossing, which are ubiquitous in brain white matter. The present article has introduced SMT to disentangle the per‐axon diffusion process from the fiber orientation distribution. This method does not make use of prior knowledge about how the fibers are oriented inside a voxel, which is normally not available in advance. Here we have demonstrated SMT in the cerebral white matter of a live human subject and provided quantitative maps of the voxel‐averaged diffusion coefficients $\lambda_{∥}$ and $\lambda_{⊥}$ parallel and perpendicular to the axons in a clinically feasible manner. In particular, there is no need for complex gradient waveforms with multiple gradient pulses 23 or magic‐angle spinning 24 to recover microscopic diffusion anisotropy as long as the local structure is axially symmetric, which is typically the case in nerve tissue. SMT just requires a conventional pulse sequence featuring two or more *b*‐shells and a uniform sampling of the gradient directions, which is well supported by the scanner vendors.

In this work, the response function is based on a second‐order approximation of the microscopic diffusion process inside the axons and in their surroundings (including glial cells and extracellular space). The transverse diffusion coefficient $\lambda_{⊥}$ is a function of the fiber caliber, the degree of myelination, and the extra‐axonal space, yet the exact relationship between $\lambda_{⊥}$ and the fiber microgeometry remains elusive. As we have made only general assumptions about the per‐axon signal, SMT can be easily extended to more complex response functions offering direct information on the axon microanatomy. The presented framework provides a blueprint for the recovery of these axon‐specific tissue features, separating them from the effects due to fiber dispersion and crossing. The key point is that the spherical mean of the diffusion signal (6) depends only on the biophysical model for a small fiber section, but not on the intravoxel axon orientation distribution. Even though brain tissue has a complex directional structure, model development is here simplified to finding a parametric response function linking the diffusion signal of an axonal segment to the underlying microstructure. Obviously, complex impulse responses require more experimental data for the robust estimation of their model parameters, which may collide with the tight time constraints imposed by human scans. Hence, the second‐order approach adopted in this work seems to offer a good compromise.

Once the impulse response has been reconstructed voxel by voxel, the estimation of the axon orientation distribution allows us to quantify fiber dispersion and crossing accurately. These benefits demonstrate the superiority of the general approach over alternative techniques, as the developed framework provides access to the axon microanatomy and a full description of the orientational architecture in a disentangled form. The orientational invariance of the per‐axon biomarkers is particularly advantageous for comparisons between subjects, as the neural circuitry is characterized by a high interindividual variability. Moreover, we expect quantifiable changes of the microscopic diffusion process in the diseased brain relative to healthy subjects. In contrast to previous work based on the standard diffusion tensor model 32, SMT is capable of discriminating structural tissue alterations from fiber orientation dispersion, which potentially improves the sensitivity and/or specificity of image‐derived parameters for diseases that directly or indirectly affect white matter. Lastly, the noninvasive quantification of microscopic diffusion anisotropy can be beneficial beyond the diagnosis of neurological conditions. For instance, SMT may be used for the discrimination of different types of tissue, for example, malignant (cancerous) and benign tumors, that appear directionally isotropic on a macroscopic scale.

To demonstrate the in vivo recovery of the per‐axon diffusion coefficients, we analyzed a dataset featuring two *b*‐shells of moderate diffusion weighting with 75 and 76 uniformly distributed gradient directions. The measurement time was about 36 min for full brain coverage with 1.5 mm isotropic voxel resolution. The scan duration can be greatly reduced to less than 5 min per subject if a more economical gradient scheme (e.g., 25 directions per *b*‐shell as in Fig. 7) is used, a lower voxel resolution is chosen, and the images are acquired with single phase‐encoding polarity, which facilitates the immediate applicability in the clinical practice. Future work will study the question of which experiment design offers the highest sensitivity to the target parameters within a given time budget on a standard MRI scanner 52. Partial volume effects due to cerebrospinal fluid contamination may be eliminated by adding a fluid attenuated inversion recovery sequence.

## Supporting information


**Figure S1.** These plots map (from top to bottom) the longitudinal and transverse microscopic diffusion coefficients $\lambda_{∥}\;\mathrm{and}\;\lambda_{⊥}$ as well as the per‐axon fractional anisotropy. The results are shown for subjects in the age group between 25 and 35 years taken from the HCP Lifespan data. Subject 1 is used in the main text and included for comparison. The estimated parameters have factored out the effects due to the intra‐voxel fibre orientation distribution. The figure demonstrates the consistency and reproducibility of SMT‐based microscopic diffusion anisotropy imaging.
