# Supplementary material for: Quantitative mapping of the per‐axon diffusion coefficients in brain white matter
Source: Magn Reson Med. 2015 May 13;75(4):1752–63. doi: 10.1002/mrm.25734 (PMC4975722; doi:10.1002/mrm.25734)
Supplement: Supplementary file 1 — Figure S1. These plots map (from top to bottom) the longitudinal and transverse microscopic diffusion coefficients λ∥ and λ⊥ as well as the per‐axon fractional anisotropy. The results are shown for subjects in the age group between 25 and 35 years taken from the HCP Lifespan data. Subject 1 is used in the main text and included for comparison. The estimated parameters have factored out the effects due to the intra‐voxel fibre orientation distribution. The figure demonstrates the consistency and reproducibility of SMT‐based microscopic diffusion anisotropy imaging. [file MRM-75-1752-s001.pdf]

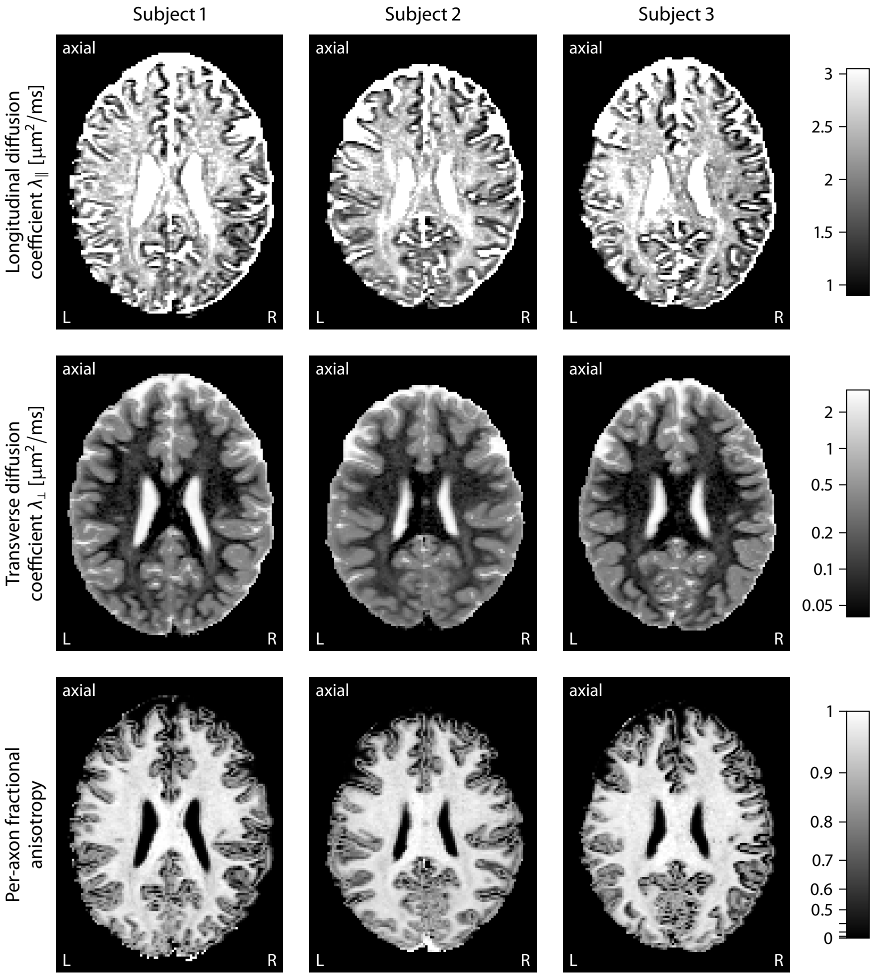

Supporting Figure S1: These plots map (from top to bottom) the longitudinal and transverse microscopic diffusion coefficients $\lambda_\parallel$ and $\lambda_\perp$ as well as the per-axon fractional anisotropy. The results are shown for subjects in the age group between 25 and 35 years taken from the HCP Lifespan data. Subject 1 is used in the main text and included for comparison. The estimated parameters have factored out the effects due to the intra-voxel fibre orientation distribution. The figure demonstrates the consistency and reproducibility of SMT-based microscopic diffusion anisotropy imaging.
